# Perception of medical students’ understanding of psychiatry as a future career in Saudi Arabia

**DOI:** 10.1192/bji.2025.10057

**Published:** 2026-02

**Authors:** Hassan Ali Alradhi, Hassan Mohammed Alturaiki, Zainb Jasim Alqurain, Nouf Ahmed Alalmaei, Safiah Ahmed Alamer, Hussain Khalifa Aljumah, Ali Alsaad

**Affiliations:** 1 Medical Student, Department of Medicine, College of Medicine, King Faisal University, Al-Ahsa, Saudi Arabia; 2 MBBS, Medical Intern, Department of Medicine, College of Medicine, King Faisal University, Al-Ahsa, Saudi Arabia; 3 Medical Student, College of Medicine and Surgery, Batterjee Medical College, Jeddah, Saudi Arabia; 4 Medical Student, Department of Medicine, College of Medicine, King Faisal University, Al-Ahsa, Saudi Arabia; 5 MBBS, Medical Intern, Department of Medicine, College of Medicine, King Faisal University, Al-Ahsa, Saudi Arabia; 6 Medical Student, Department of Medicine, College of Medicine, King Faisal University, Al-Ahsa, Saudi Arabia; 7 MD, Associate Professor, Department of Medicine, College of Medicine, King Faisal University, Al-Ahsa, Saudi Arabia

**Keywords:** Medical student, psychiatry, attitudes, career choice, Saudi Arabia

## Abstract

**Background:**

Mental health disorders, including depression and anxiety, pose significant public health challenges globally, and in Saudi Arabia. Despite this, psychiatry suffers from a critical shortage of specialists. This study investigates factors influencing medical students’ career decisions in regard to psychiatry, aiming to enhance understanding of, and address workforce deficiencies in, mental healthcare.

**Aims:**

This study aims to investigate the factors influencing medical students’ decision to choose psychiatry as a future career.

**Method:**

This cross-sectional study conducted an online survey among Saudi Arabian medical students from 28 December 2023 to 28 April 2024, employing validated questions refined through pilot testing. Participant selection included male and female students across preclinical and clinical stages, excluding non-medical students and those outside Saudi Arabia.

**Result:**

This study explores the perceptions and interest of 430 medical students in Saudi Arabia regarding psychiatry as a career. The majority were female (69.3%), with most in their preclinical years (60.2%). Key findings include limited personal connections to psychiatry (9.5% with a family psychiatrist), and primarily influenced by medical school (55.3%) and social media (42.1%). While 65.1% perceive psychiatry as mentally demanding, uncertainties exist about career prospects and stigma persists (39.1%). Gender differences were observed, with more females (34.6%) than males (22.7%) interested in psychiatry (*P* = 0.014). Early inspiration in medical education significantly increased interest (*P* = 0.001).

**Conclusion:**

Early exposure, personal connections and gender-specific factors significantly influence medical students’ interest in psychiatry as a career. Integrating psychiatry education early in medical curricula and addressing stigma are critical for fostering positive perceptions and attracting diverse students to the field.

Psychiatry is a field of medicine that focuses on the diagnosis, treatment and prevention of mental illnesses. These are prevalent, significant health illnesses that have a detrimental effect on a person’s capacity to live a fruitful, meaningful life and build profound relationships with family, friends and other people.^1^ As a physician, the psychiatrist can perform the necessary medical and psychological tests to evaluate the case, reach a proper diagnosis and choose the best treatment plan.^2^

According to a 2019 Global Burden of Diseases (GBD) study, mental illness – particularly depression and anxiety – ranks among the top 10 primary causes of disease burden, with an estimated incidence of 654.8 million cases in 1990 and 970.1 million in 2019, representing a 48.1% increase over these 3 decades.^3^ In the absence of medical intervention, mental health disorders can have devastating impacts on both the individual and society. Suicide, unwarranted incarceration, substance misuse, homelessness, undeserved handicapping and low quality of life are all consequences of untreated mental health disorders.^4^

The Eastern Mediterranean Region (EMR) of the World Health Organization (WHO), which includes Saudi Arabia, suffers greatly from mental illnesses, with depression and anxiety being the most common illnesses, particularly in nations affected by conflicts and other adverse events.^5^ In Saudi Arabia, the prevalence and risk of depression are relatively high compared with other Gulf countries, affecting more than a third of the population, with females being more at risk than males.^6^ Moreover, this risk has increased following the COVID-19 pandemic.^7^ As people have struggled with the ensuing health, social and economic consequences, their mental health has suffered significantly. The pandemic triggered or exacerbated much more serious mental health issues. According to WHO, many people have reported psychological suffering, including symptoms of sadness, anxiety or post-traumatic stress.^8^

Unexpectedly, despite the high prevalence of mental illness, psychiatry has one of the most significant shortages among medical disciplines.^9^ According to the Saudi Ministry of Health (MOH), there are only 1565 psychiatrists (486 per 100 000 population), amounting to 1.5% of all physicians (105 332), including those within the MOH, other public sectors and private sectors.^10^ This is significantly lower than the 1306 psychiatrists per 100 000 persons reported in high-income countries.^11^ Saudis in need of mental health treatment would therefore suffer from a lack of psychiatrists. Therefore, training of psychiatrists is crucial and, as numerous studies have consistently demonstrated, psychiatry is not the most popular speciality among medical students worldwide.^12,13^

The choice to become a psychiatrist is impacted by numerous considerations, one of which is the individual’s perception of the profession.^9^ One study of international medical students’ opinions on psychiatry found an unfavourable perception of the profession.^13^ These students viewed psychiatry as not being part of mainstream medicine, having poor public status and indicating a lack of academic strength in those who show interest in the profession.^14^ Nevertheless, research has indicated that being exposed to psychiatric services can potentially foster a favourable outlook on psychiatry, and the quality of experience of, and exposure to, psychiatry as a medical undergraduate in medical school could be the most crucial factor influencing recruitment into the field of psychiatry.^15–17^

Stigma, encompassing cultural, personal and professional aspects, has the potential to adversely influence perceptions of psychiatry and individuals with mental health conditions.^18^ Medical students’ poor perceptions of psychiatry have been linked to stigma surrounding mental illness, because they hold stigmatising beliefs about mental illness that are comparable to those expressed in public surveys.^19^ For instance, subjects with mental illness tend to be viewed as dangerous, unstable, unreliable and unappealing.^20^

This study seeks to explore medical students’ perceptions of the field of psychiatry, which is critical given their future role in healthcare provision. It is essential to consider that unfavourable views of psychiatry can significantly impact the well-being of individuals receiving psychiatric care.^17^ To the best of our knowledge, few studies have been conducted in Saudi Arabia to explore the factors that influence medical students in choosing psychiatry as a future career, and their views of the speciality.

## Method

### Study population

This research utilised a descriptive cross-sectional methodology, conducted in various Saudi Arabian cities, from 28 December 2023 to 28 April 2024. The primary data collection tool was an online questionnaire, aimed at collecting demographic information, among other data. The questionnaire was distributed to medical students primarily via email and through WhatsApp groups, which are widely used among the student community for communication and information sharing. The survey link was also promoted by online platforms. While no incentives were offered, the importance of contributing to advancements in medical education was emphasised, resulting in a strong participation rate. Initially the survey underwent a validation process via a pilot study, which involved the formulation of pertinent questions, consultation with experts and distribution of a preliminary version to ten researchers for feedback, leading to subsequent modifications based on their suggestions.

In terms of participant selection, the study included both male and female medical students, encompassing those in both preclinical and clinical stages, from all regions of Saudi Arabia. Additionally, individuals with relatives working in the psychiatric field were also included. On the other hand, the study excluded non-medical students from other colleges, as well as residents and consultants and those residing outside Saudi Arabia.

Data collection was carried out using an online Excel sheet linked to the survey, emphasising a strong commitment to preserving the privacy and confidentiality of participants’ information. Data analysis was performed using the IBM Statistical Package for the Social Sciences (SPSS; Armonk, New York, NY, USA) for Windows 10 (version 2021) and Microsoft Excel version 2021.

### Ethical issues

This study was conducted without any interventions, experiments or adjustments to the patient’s treatment plans. The study adhered to standard practices and was anticipated to present no significant risk or burden to participants. Participants were thoroughly informed of the study’s purpose at the start of the questionnaire, and their choice to continue and complete the survey was taken as an indication of their informed written consent.

The authors declare that this study was conducted within standard practice and posed no negligible risk or burden. Ethical approval for this research was secured from King Faisal University, Al Ahsa, Saudi Arabia (no. KFU-REC-2023-DEC-ETHICS1806).

### Data analysis

The data were collected, reviewed and then input to IBM SPSS Statistics for Windows version 18.0 (IBM Corp., Armonk, NY, USA; https://www.ibm.com/products/spss-statistics). All statistical methods used were two-tailed, with an alpha level of 0.05 and considering significance at *P* ≤ 0.05. Descriptive analysis was done by prescribing frequency distribution and percentage for study variables, including student’s age, gender and academic qualifications. In addition, medical students’ awareness and exposure for psychiatry speciality, their perception and understanding of psychiatry speciality, stigma and satisfaction, residency and psychiatry as a future career were also tabulated. Medical students’ interest in psychiatry as their future specialties was graphed. Cross-tabulation was used to show factors associated with medical students’ interest in psychiatry as a future speciality, using the Pearson chi-square test for significance and the exact probability test if there were small frequency distributions.

## Results

A total of 430 medical students were included, with age ranging from 18 to 26 years and a median of 21. A total of 298 (69.3%) were female, most of whom (410, 95.3%) were Saudi. A total of 413 (96%) were unmarried. In regard to academic year, 259 (60.2%) were in their preclinical years (1st to 3rd), 163 (37.9%) in their clinical years (4th to 6th) and 8 (1.9%) were medical interns (Table 1).

**Table 1:** Demographics of participants (*N* = 430)

Demographic data	*n*	%
Age, years		
18–19	63	14.7
20–21	234	54.4
22–26	133	30.9
Gender		
Male	132	30.7
Female	298	69.3
Nationality		
Saudi	410	95.3
Non-Saudi	20	4.7
Marital status		
Unmarried	413	96.0
Married	17	4.0
Academic year		
1st	25	5.8
2nd	78	18.1
3rd	156	36.3
4th	107	24.9
5th	33	7.7
6th	23	5.3
7th	8	1.9
Academic phase		
Preclinical	259	60.2
Clinical	163	37.9
Medical intern	8	1.9

A total of 389 (90.5%) students did not have a family member working as a psychiatrist. In regard to the original source of their awareness of psychiatry, the most frequently reported were medical school (55.3%), social media (42.1%), hospital training (23.3%), mentor (21.9%) and friends (19.8%). When asked about when they were first inspired about psychiatry, the most frequent responses were before medical school (23.3%), at year 2 of medical school (10.5%) and at year 3 of medical school (7.2%), while 222 (51.6%) were not inspired about psychiatry (Table 2).

**Table 2:** Medical students’ awareness of, and exposure to, the psychiatry speciality

Questions put to students	*n*	%
Do you have a family family working as a psychiatrist?		
Yes	41	9.5
No	389	90.5
Original source of perception about psychiatry?		
Medical school	238	55.3
Social media	181	42.1
Hospital training	100	23.3
Mentor	94	21.9
Friends	85	19.8
Family member	83	19.3
Other	74	17.2
When were you first inspired about psychiatry?		
1st year at medical school (preparatory year)	12	2.8
2nd year at medical school	45	10.5
3rd year at medical school	31	7.2
4th year at medical school	8	1.9
5th year at medical school	10	2.3
6th year at medical school	2	0.5
Before medical school	100	23.3
I am not inspired about psychiatry	222	51.6

A total of 280 (65.1%) students considered psychiatry a mentally demanding speciality (i.e. perceived it as psychologically draining or exhausting); 188 (43.7%) reported that the personality and attitude of a psychiatric professional are not significantly different from those of any other physician; but 136 (31.6%) considered that such a personality is seen as strange behaviour. In addition, 288 (67%) thought that there are learning and academic opportunities in psychiatry, and 210 (48.8%) viewed psychiatry as an interesting speciality; 148 (45.1%) rated their level of interest as 1–4 out of 10, and 51 (15.5%) rated it as 8–10. A total of 143 (33.3%) rated psychiatry as 4 out of 5 in terms of challenge, and 103 (24%) rated it as 3 out of 5, based on a Likert scale where 1 represents ‘not challenging at all’ and 5 represents ‘extremely challenging’. This scale assessed perceived emotional intensity, cognitive demands and the complexity of patient interactions inherent in psychiatry. In regard to stigma surrounding the profession of psychiatry, 168 (39.1%) considered that it to have a bad reputation and poor prestige, but 213 (49.5%) reported an acceptable satisfaction rate, 42 (9.8%) showed high satisfaction and 64 (14.9%) reported low satisfaction (Table 3).

**Table 3:** Medical students’ perception and understanding of psychiatry speciality, stigma and satisfaction

Questions put to students	*n*	%
Do you think psychiatry is a mentally demanding speciality?		
Yes	280	65.1
No	44	10.2
Not sure	106	24.7
What are your opinions about the personality and attitude of a psychiatric professional?		
Attitude is not significantly different from any other physician	188	43.7
No relationship between speciality and personality	106	24.7
Personality is seen as a strange behaviour	136	31.6
What do you think about psychiatry?		
Not sure	111	25.8
Opportunities for learning, and also academic opportunities	288	67.0
There are no learning or academic opportunities	31	7.2
What is your opinion regarding the interest you have?		
Interested	210	48.8
Not interested	102	23.7
Not sure	118	27.4
How would you rate your current level of interest in psychiatry as a speciality, on a scale from 1 to 10?		
1–4	148	45.1
5–7	129	39.3
8–10	51	15.5
How challenging is psychiatry as a speciality, on a scale of 1–5?		
1–2	31	7.2
3	103	24.0
4	143	33.3
5	72	16.7
Not sure	81	18.8
What negative perceptions or stereotypes do you believe exist regarding the field of psychiatry?		
Not sure	147	34.2
It has a good reputation and level of prestige	115	26.7
It has a bad reputation and level of prestige	168	39.1
How do you rate the level of satisfaction in psychiatry?		
Low	64	14.9
Acceptable	213	49.5
High	42	9.8
Not sure	111	25.8

A total of 184 (42.8%) students were of the opinion that their income following completion of psychiatric residency training would be acceptable, but 140 (32.6%) were unsure. In addition, 180 (41.9%) thought that the rate at which applicants are accepted into residency programmes was average, but 161 (37.4%) were unsure. In regard to on-call responsibilities and scheduling for psychiatric residents, 187 (43.5%) considered this acceptable while 200 (46.5%) were unsure. With regard to opinions on the probable duration of psychiatric residency training, 212 (49.3%) of the students stated 4 years, 94 (21.9%) stated 3 years and 84 (19.5%) stated 5 years or more (Table 4).

**Table 4:** Psychiatric speciality residency perception and understanding among medical students

Questions put to students	*n*	%
What are your opinions regarding income level following completion of psychiatric residency training?		
Low	29	6.7
Acceptable	184	42.8
High	77	17.9
Not sure	140	32.6
What are your thoughts on how well applicants are accepted into residency programmes?		
Easy	39	9.1
Average	180	41.9
Difficult	50	11.6
Not sure	161	37.4
What are your thoughts regarding on-call responsibilities and scheduling for psychiatric residents?		
Unacceptable	43	10.0
Acceptable	187	43.5
Not sure	200	46.5
What do you consider to be the duration of psychiatric residency training?		
2 years	40	9.3
3 years	94	21.9
4 years	212	49.3
5 years	84	19.5

A total of 162 students (37.7%) considered the career opportunities in the field of psychiatry following residency as good, and 141 (32.8%) as limited. Likewise, 205 (47.7%) were of the opinion that there were good opportunities for research in psychiatry while 232 (54%) were unsure about pursuing a fellowship in psychiatry; 99 (23%) thought that there were many fellowships available in psychiatry, but an identical number thought the opposite. In regard to the duration of patient–physician relationships in the field of psychiatry, 245 (57%) thought it should be a lifetime relationship while 134 (31.2%) were unsure. In addition, 258 (60%) were of the opinion that there is a good life–work balance in psychiatry, but 113 (26.3%) were unsure (Table 5).

**Table 5:** Medical students’ perception and understanding of psychiatry as a future career

Questions put to students	*n*	%
What do you think about the career opportunities in the field of psychiatry following residency?		
Good	162	37.7
Limited	141	32.8
Not sure	127	29.5
What do you think about the availability of research opportunities in psychiatry?		
Good	205	47.7
Limited	98	22.8
Not sure	127	29.5
What are your thoughts on pursuing a fellowship in the field of psychiatry?		
Not sure	232	54.0
There are many fellowships available in psychiatry	99	23.0
There are not many fellowships available in psychiatry	99	23.0
What are your thoughts on the duration of patient–physician relationships in the field of psychiatry?		
Lifetime relationship	245	57.0
Not sure	134	31.2
One-time relationship	51	11.9
What do you think about the life–work balance in psychiatry?		
Not sure	113	26.3
There is a balance	258	60.0
There is no balance	59	13.7

When asked about their interest in a career in psychiatry, 34.6% of female students and 22.7% of male students replied in the affirmative, with a recorded statistical significance of *P* = 0.014. In addition, 50% of non-Saudi students showed an interest in psychiatry as a future career compared with 30% of Saudi students (*P* = 0.049). A proportion of 73.2% of students who had a family member working as a psychiatrist showed an interest in the speciality, compared with 26.5% who did not (*P* = 0.001). Likewise, 87.5% of students who were inspired about the speciality in their 4th medical year showed an interest, compared with 3.2% of those who were not inspired (*P* = 0.001) (Table 6).

**Table 6:** Factors associated with medical students’ interest in psychiatry as a future speciality

Students’ demographics	Are you interested in psychiatry as a future speciality?				*P*-value
	Yes		No		
	*n*	%	*n*	%	
Age, years					0.989
18–19	20	31.7	43	68.3	
20–21	72	30.8	162	69.2	
22–26	41	30.8	92	69.2	
Gender					0.014*
Male	30	22.7	102	77.3	
Female	103	34.6	195	65.4	
Nationality					0.049*
Saudi	123	30.0	287	70.0	
Non-Saudi	10	50.0	10	50.0	
Marital status					0.142
Unmarried	125	30.3	288	69.7	
Married	8	47.1	9	52.9	
Academic phase					0.361
Preclinical	85	32.8	174	67.2	
Clinical	47	28.8	116	71.2	
Medical intern	1	12.5	7	87.5	
Do you have a family member working as a psychiatrist?					0.001*
Yes	30	73.2	11	26.8	
No	103	26.5	286	73.5	
When were you first inspired about psychiatry?					0.001*^a^
1st year at medical school (preparatory year)	8	66.7	4	33.3	
2nd year at medical school	21	46.7	24	53.3	
3rd year at medical school	20	64.5	11	35.5	
4th year at medical school	7	87.5	1	12.5	
5th year at medical school	5	50.0	5	50.0	
6th year at medical school	1	50.0	1	50.0	
Before medical school	64	64.0	36	36.0	
I am not inspired about psychiatry	7	3.2	215	96.8	
Original source of perception about psychiatry?					0.705
Medical school	70	29.4	168	70.6	
Social media	59	32.6	122	67.4	
Mentor	32	34.0	62	66.0	
Hospital training	28	28.0	72	72.0	
Friends	29	34.1	56	65.9	
Family member	31	37.3	52	62.7	
Other	24	32.4	50	67.6	

*P*, Pearson’s *X* ^2^ test.

a Exact probability test.

* *P* < 0.05 considered significant.

## Discussion

This study aimed to explore the awareness, perceptions and interest of medical students in Saudi Arabia towards psychiatry, while also examining factors that influence their potential choice of psychiatry as a future career. This research, which involved 430 medical students, has revealed several significant findings regarding their perceptions of, and interest in, psychiatry.

The findings indicate that the majority of students perceive psychiatry as a mentally demanding speciality yet are uncertain about its career prospects and research opportunities. Interestingly, the influence of personal connection to the field through family members and the timing of exposure to psychiatry during medical education appear to be significant determinants of interest. This underscores the importance of early exposure and personal connections in shaping students’ perceptions and career choices. Moreover, only a small fraction of students (9.5%) had a family member working as a psychiatrist, suggesting limited direct exposure to the profession. The primary sources of their perceptions about psychiatry were medical school (55.3%) and social media (42.1%), demonstrating that educational settings and online platforms play significant roles in shaping students’ views. A majority (65.1%) viewed psychiatry as a mentally demanding speciality. However, there was a disparity in how they viewed the personality of psychiatrists and the stigma associated with the profession: 43.7% believed that a psychiatrist’s personality is not significantly different from that of other physicians, yet 39.1% acknowledged the presence of stigma surrounding the speciality. Students held mixed views regarding their career prospects in psychiatry: 42.8% considered the income post-residency as acceptable, yet there was considerable uncertainty about residency acceptance rates and on-call responsibilities. Regarding career opportunities post-residency, 37.7% viewed these as good but a similar proportion (32.8%) considered them to be limited.

### Gender differences in shaping interest in psychiatry

Our study indicates a moderate level of overall interest in psychiatry. Importantly, we observed a discernible inter-gender difference in interest, with a significantly higher percentage of female students expressing a desire to pursue psychiatry compared with their male counterparts. This finding indicates that gender may play a significant role in determining speciality choice, underscoring the profound connection between gender and the selection of a medical profession. Building on this observation, our results resonate with the findings from previous research. According to Christov-Moore et al, it is suggested that female individuals may exhibit a more favourable attitude towards professions requiring high levels of empathy, such as psychiatry, due to their statistically higher empathy levels.^21^ The parallel drawn between our findings and those of Christov-Moore et al reinforces the notion that gender-specific factors, including societal norms and inherent interpersonal skills, significantly influence medical students’ speciality preferences. This is particularly evident in fields such as psychiatry, where a deep understanding and empathetic patient interaction are crucial.

### Comparison with existing literature

Several studies have addressed the factors that impact medical students’ interest in psychiatry and their perceptions of the speciality. By reaffirming and providing new insights into existing research, our study highlights the critical role of early exposure, personal connections and gender-specific factors in shaping medical students’ perceptions of, and interest in, psychiatry as a potential career path. For instance, research by Agyapong et al demonstrated that exposure to psychiatry during medical school significantly increased students’ interest in pursuing a career in the field, a finding consistent with our own that emphasises the importance of early exposure to psychiatry in shaping students’ perceptions and career choices.^22^ Additionally, the study by Seow et al suggested that personal connections, such as having a family member working as a psychiatrist, can influence students’ interest in psychiatry, which aligns with our finding that personal connections play a crucial role in shaping students’ perceptions and interest.^23^

Furthermore, research by Farooq et al revealed that female medical students were more inclined to choose psychiatry as a career compared with their male counterparts, a trend consistent with our observation of a higher percentage of female students expressing interest in pursuing a career in psychiatry.^18^

In summary, our research contributes to the existing literature by confirming the significance of early exposure, personal connections and gender in shaping medical students’ interest in psychiatry as a career choice. The findings from this study have significant implications for the field of psychiatry. First, the importance of early exposure to psychiatry in medical school cannot be understated. Medical educators should consider integrating psychiatry teaching and clinical rotations early in the curriculum, to foster interest and create positive perceptions. Additionally, efforts should be made to address the stigma associated with the field and highlight the career prospects and research opportunities available in psychiatry. Moreover, the gender differences in regard to interest that were detected suggest the need for targeted interventions to attract more male students to the speciality. Understanding these factors can help inform strategies to cultivate a diverse and well-rounded workforce in psychiatry.

### Limitations and future directions

While this study provides important insights, it is essential to recognise certain limitations. First, the sample of 430 medical students may not fully capture the views of all medical students across Saudi Arabia, which could limit the generalisability of our findings. Additionally, because data were collected through an online, self-reported survey, there is a chance of response bias. The cross-sectional nature of the study further restricts our ability to infer causal relationships or track changes in students’ perceptions and interests over time. Furthermore, the sample selection focused on students in particular academic phases and those with family connections to psychiatry, potentially introducing sampling bias that could have affected the observed interest levels in psychiatry. Future studies should consider a longitudinal design to track a cohort of medical students over time, to observe how their views on psychiatry develop throughout their education and into the early stages of their careers. This approach could include multiple survey points, such as at the start of medical school, following psychiatry rotations and post-graduation, to assess how exposure, clinical experiences and external influences shape their interest in the speciality.
